# An Open-Source Platform for Human Pose Estimation and Tracking Using a Heterogeneous Multi-Sensor System

**DOI:** 10.3390/s21072340

**Published:** 2021-03-27

**Authors:** Ashok Kumar Patil, Adithya Balasubramanyam, Jae Yeong Ryu, Bharatesh Chakravarthi, Young Ho Chai

**Affiliations:** Virtual Environments Lab, Graduate School of Advanced Imaging Science, Multimedia and Film, Chung-Ang University, Seoul 06974, Korea; ashokpatil03@cau.ac.kr (A.K.P.); adithyab@cau.ac.kr (A.B.); fbwodud92@naver.com (J.Y.R.); bc05@cau.ac.kr (B.C.)

**Keywords:** human pose estimation, detection, tracking, multi-sensor, heterogeneous sensor, sensor fusion, lidar sensor, inertial sensor

## Abstract

Human pose estimation and tracking in real-time from multi-sensor systems is essential for many applications. Combining multiple heterogeneous sensors increases opportunities to improve human motion tracking. Using only a single sensor type, e.g., inertial sensors, human pose estimation accuracy is affected by sensor drift over longer periods. This paper proposes a human motion tracking system using lidar and inertial sensors to estimate 3D human pose in real-time. Human motion tracking includes human detection and estimation of height, skeletal parameters, position, and orientation by fusing lidar and inertial sensor data. Finally, the estimated data are reconstructed on a virtual 3D avatar. The proposed human pose tracking system was developed using open-source platform APIs. Experimental results verified the proposed human position tracking accuracy in real-time and were in good agreement with current multi-sensor systems.

## 1. Introduction

Many studies have investigated accurately estimating and tracking three-dimensional (3D) objects in real-time using single or multiple sensor systems [1,2,3]. Tracking 3D human motion has made significant progress recently due to advanced object tracking sensor availability, and has become a useful technique in various applications, such as human–computer interaction (HCI), activity recognition, virtual reality, fitness training, healthcare, and rehabilitation [4]. Significant milestones have been achieved for tracking human pose using depth, inertial, vision, light detection and ranging (lidar) sensor systems, and more recently, heterogeneous multi-sensor systems [5].

In particular, vision-based human motion tracking has been widely studied. Full body 3D pose reconstruction from single view images is difficult and suffers from the ill-posed problem, compared with two-dimensional or 3D pose estimation from multiple views. Additional constraints on kinematics and movement are typically employed to resolve inherent ambiguity in monocular images [6].

Depth sensors have become widespread due to ease of use, availability of open-source tools and communities, such as Microsoft Kinect, that automatically infer 3D joint positions from single depth data. Depth sensors convert depth data into RGBZ data, which helps detect human joints [7] and extract rotational information from the skeletal structure. However, the methods suffer from occlusion [8]. Although multiple depth sensors strategically positioned in the environment [9] can reduce body occlusion, they cannot fully compensate for it.

Inertial sensors, also known as inertial measurement units (IMUs), are commonly rigidly attached to an object to help track or estimate position and orientation information [10]. IMU sensors have been applied to a greater number of application areas, including pose estimation for robotics, autonomous vehicles [11], and human motion tracking [1] and visualization [12]. However, although IMU sensors are accurate over short periods, they suffer from occlusions and drift over longer periods [13], and hence, are commonly combined with other sensors.

Three-dimensional lidar sensor applications have expanded dramatically over the last few decades [14], including robotics, autonomous vehicles, HCI, and human pose detection and tracking. Lidar sensors provide wide angle and long distance laser scan data as intensity point clouds. Point cloud data are denser at near distance and sparser as distance increases. Long distance data are not usually affected by lighting conditions, hence the data can be very accurate. However, human detection and tracking remains challenging in lidar data, particularly when the tracked person or object is too near or too far from the lidar sensor. Lidars are often employed as single sensors [15] or fused with other sensors, such as IMUs [2,16] and/or vision sensors [17].

**Figure 1:**
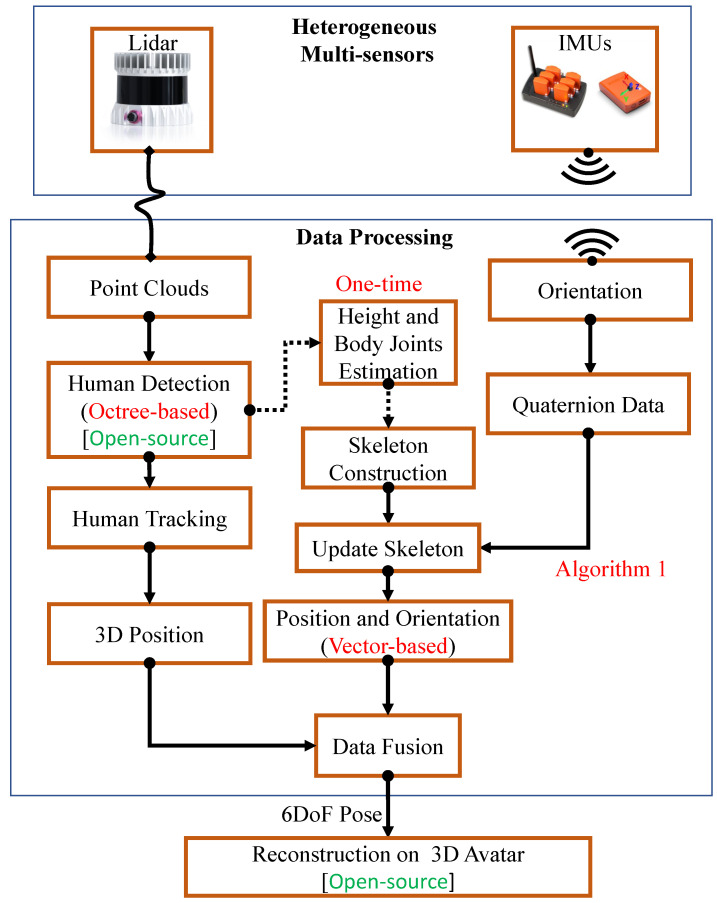
Proposed system workflow.

**Figure 2:**
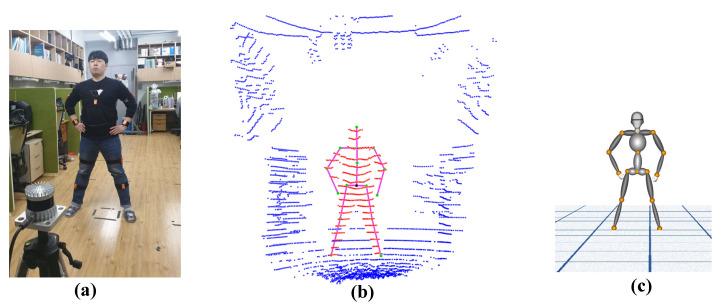
Proposed human tracking system overview: (**a**) Heterogeneous sensing experimental setup, (**b**) Detected user in lidar data and skeleton construction, (**c**) Reconstruction on 3D Avatar.

The proposed system provides a more feasible and robust system for human pose estimation with accurate detection, tracking, and reconstruction on a virtual avatar using multiple sensors (Lidar and IMUs) on an open-source platform. Figure 1 shows the proposed system workflow and Figure 2 shows an overview for pose tracking.

The proposed system proceeds as follows.

- Detect human body information from background lidar data using Octree based change detection
- Estimate human height and skeletal parameters
- Track position and orientation using multiple heterogeneous sensors and
- Reconstruct human motion on a 3D Avatar.

The remainder of this paper is structured as follows. Section 2 discusses related studies on human pose tracking. Section 3 details the proposed heterogeneous multi-sensor system for human pose estimation and tracking, and Section 4 evaluates the proposed system experimentally. Finally, Section 5 summarizes and concludes the paper.

## 2. Related Work

Many previous studies have proposed IMU-based human motion tracking techniques and methodologies. IMU tracking provides accurate orientation when the sensors are attached to a rigid body from the object of interest. However, occlusion and drift occur for continuous measurement over long periods. Filippeschi et al. [18] discussed inertial sensor issues and advantages, then compared five IMU tracking techniques for motion reconstruction on human arm motion with a commercially available motion tracking system as ground truth. Fuyang et al. [19] discussed IMU-based approaches’ strengths and weaknesses.

Qiu et al. [20] proposed a multi-sensor fusion methodology to address challenges from pedestrian dead reckoning. They fused Xsens IMU sensor and Vicon optical motion capture system data to obtain 3D orientation and position, and employed an extended Kalman filter to minimize errors induced from magnetic disturbance. Li et al. [21] proposed an optical–inertial data fusion scheme to provide resistance for optical human body joint data and rectify error accumulated in inertial data, providing long term drift-free operation. Jilliam et al. [22] proposed distance transformation and principal component analysis based human pose estimation using multi-view systems comprising multiple depth cameras. Multi-point cloud video sequences were used to represent the human body external surface, limited to known human body proportions. Yan et al. [23] proposed a hierarchical optimized Bayesian sensor fusion framework to calculate voxel occupancy probability and hence realize a markerless human motion tracking system, and compared their results against marker-based motion capture systems.

Three-dimensional human pose tracking and estimation generally employs vision, IMU, or heterogeneous sensor fusion based methods. Although vision systems are widely used to capture joint positions, they have major limitations related to occlusion and illumination changes. IMU-based motion capture systems can acquire accurate bone segment orientation, but poorly estimate joint positions and suffer from sensor drift. On the other hand, heterogeneous sensor fusion methods can effectively combine the two modalities to provide greater reliability. Huang et al. [19] discussed several vision, IMU, and sensor fusion methods for 3D human pose tracking.

Charles et al. [24] proposed a real-time motion capture-system with no optical markers to fuse multi-view camera and IMU data by integrating position, orientation, pose, and acceleration. Pons-Moll et al. [25] proposed inverse kinematics and von Mises-Fisher sampling optimization to limit orientation cues from IMU and low dimensional manifold images cues on an inverse kinematic model. Trumble et al. [3] proposed a 3D convolution neural network self-learning technique to fuse volumetric and IMU data. Bone orientations acquired from IMU sensors were converted to bone joint position by adding forward kinematics, and then joint positions obtained from both sources were fused at the end of the network by fully connected layers. Marcard et al. [26] proposed a single hand-held camera and set of IMUs optimization techniques to jointly optimize vision and IMU data on a statistical body model. However, they optimized their model overall frames simultaneously, limiting its application for offline systems. Some recent works focused on human posture detection and classification in the healthcare system by integrating machine learning and deep neural network with multisensory data fusion for posture recognition  [27,28,29].

Ziegler et al. [2] proposed a system where a mobile robot equipped with a laser range finder followed a person wearing an IMU suit. They obtained accurate body postures by using the range finder to measure distances between the robot and the person’s legs, correcting for IMU drift. However, the requirement for a moving robot and laser range finder limited scan area makes this approach impractical for indoor use. Cheng et al. [30] proposed a multi-sensory fusion method open-source platform for human motion tracking, using time-of-arrival based distance ranging to correct sensor drift, and a geometrical kinematic model and maximum entropy Kalman filter for sensor fusion.

## 3. Materials and Methods

We used a multi-sensor system to gather human motion data for pose estimation, tracking, and reconstructing on a 3D avatar, comprising a single lidar and 10 IMU sensors. 3D lidar data were used to track human body position, and IMUs data to estimate orientation and position for each joint during human movement.

### 3.1. Heterogeneous Multi-Sensor Setup

**Figure 3:**
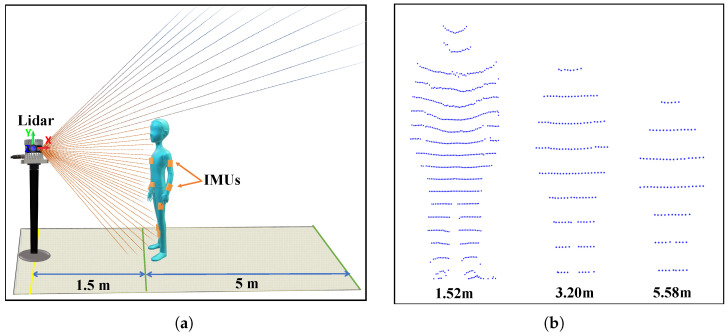
(**a**) Proposed multi-sensor system setup, (**b**) Human body tracking at different distance in indoor environment by 3D lidar.

Figure 3a shows the multi-sensor system setup employed for the experiments. The lidar sensor was placed at a fixed location, and IMU sensors were placed on 10 bone joints. Laser rays depicted in the figure represent the lidar vertical field of view (FoV). The proposed system extends our preliminary work [31], which employed a similar number of IMU sensors but two lidar sensors placed perpendicularly to track human position. A unique position tracking estimation technique is achieved in the preliminary work. Since slight inclination occurs in mounting the first lidar ($L_{1}$ in [31]) on the ceiling, the position estimation differs.

The maximum range for human body tracking is 14–17 m [32], depending on the lidar sensor specification and working environment. Figure 3b shows human detection scenarios at different distances using a lidar sensor. We used a 32 channel lidar sensor with 55 m range and ±1.5–5 cm accuracy. Lidar accuracy varies with distance from the sensor, being somewhat better within 1–15 m (±1.5 cm). Therefore, the proposed setup ensured the lidar was well within the best operating range for the indoor environment (4×8 m), with a human motion tracking area of 1.5–6.5 m distance (full tracking range in the *x* axis = 5 m), as shown in Figure 3a (green lines).

### 3.2. Height Estimation and Skeleton Parametrization

User height and bone joint locations for skeletal construction were computed before tracking the user in real-time. The user stood at optimal distance (1.5 m) from the lidar (Figure 3a) to ensure their full body was within the lidar FoV. The lidar vertical FoV is 90°, hence the user needed to be at least 1 m from the lidar for accurate height estimation—the optimal distance would be 1.5 m. In the preliminary work, the user needs to stand within the two lidars’ collective FoVs to estimate the height since the previous lidar specification has a 30° vertical FoV.

User height must be set manually for commercially available IMU sensor bodysuits for the calibration process [13,19], whereas the proposed approach estimates height from lidar point cloud data. The procedure for initial skeleton bone joints estimation was similar to the preliminary study approach [31], except for the height estimation.

Two different point cloud datasets, *P*, were acquired during calibration to detect the user from lidar data. The reference set, $P_{r}$, excluded the user, providing the background data; whereas the other point cloud, $P_{f}$, included the user in the FoV. We compared $P_{r}$ and $P_{f}$ using an Octree-based point cloud change detection algorithm [33] that filtered point cloud data ($P_{t}$) corresponding to the user (see Figure 2b).

Actual height $A_{h}$ estimation in the preliminary work required estimating the lidar slope *m* arising from employing two lidar sensors with slight inclination. However, the current setup included only a single lidar and hence *m* was not required. Ground point g(x,y,z) was estimated by computing $P_{t}$ centroid c(x,y,z) for the *x* and *z* components, and the *y* component was $max_{y}=\max\left(y\right)\in P_{r}$, i.e.,

$$g\left(x,y,z\right)=g(c_{x},P_{r}\left(max_{y}\right),z).$$

Therefore, actual height can be expressed as

$$A_{h}=\left|\left(g_{y}\right)-\left(P_{t}\left(max_{y}\right)\right)\right|,$$

The estimated $A_{h}$ gives the user actual height with an accuracy of ±2 cm. $A_{h}$ is the baseline for constructing the user’s skeleton.

**Figure 4:**
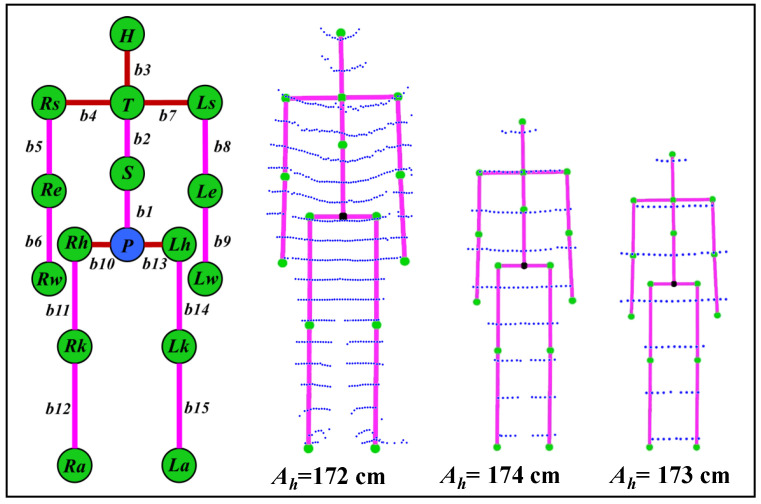
Actual height estimation and skeleton construction at different distances.

Figure 4 shows the skeleton structure with 15 segments (b1 to b15) and 16 connecting joints parameters. It also shows the estimated height of a user (real height of the user is 172 cm) and skeleton construction for the data shown in Figure 3b. Detailed skeleton parameterization and construction is given in [31].

### 3.3. Heterogeneous Pose Tracking

The above process is an initial step for calibration and configures the human skeleton. Next is a pose tracking process for locating bone joints and segments’ position and orientation in each heterogeneous sensors (Lidar and IMUs) frame in real-time. The conventional usage of IMUs in motion tracking is to estimate the relative movement of the attached bone segment in terms of position and orientation. In proposed work, the position and orientation of 10 bone segments are estimated using a vector-based method from 10 IMU sensors. The sensors must be calibrated before capturing tracking data to avoid incorrect estimation and reduce sensor drift, otherwise leading to bone segment misalignment and mismatching for the avatar in real-time. The calibration routine has one step with an attention pose.

**Figure 5:**
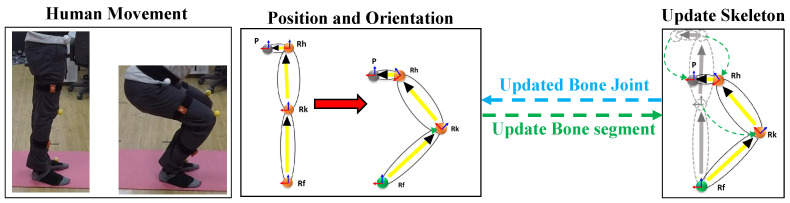
Vector-based right leg bone joint position and orientation estimation.

We considered each joint position to be a unit vector in the direction parallel to the respective bone axis in the attention pose. The orientation in the form of quaternion from the IMU sensor is multiplied to the unit vector to update the joint position. The vector-based bone joint position and orientation estimation is given in the Algorithm 1. Figure 5 shows the vector-based pose estimation from the IMU sensors and more detail is discussed in [31]. Parallelly, the position from the lidar data was estimated by detecting the user point cloud in real-time by using a similar step as discussed above in Section 3.2 for the continuous point cloud frames.
**Algorithm 1:** Vector-based position and orientation estimation in real-time.
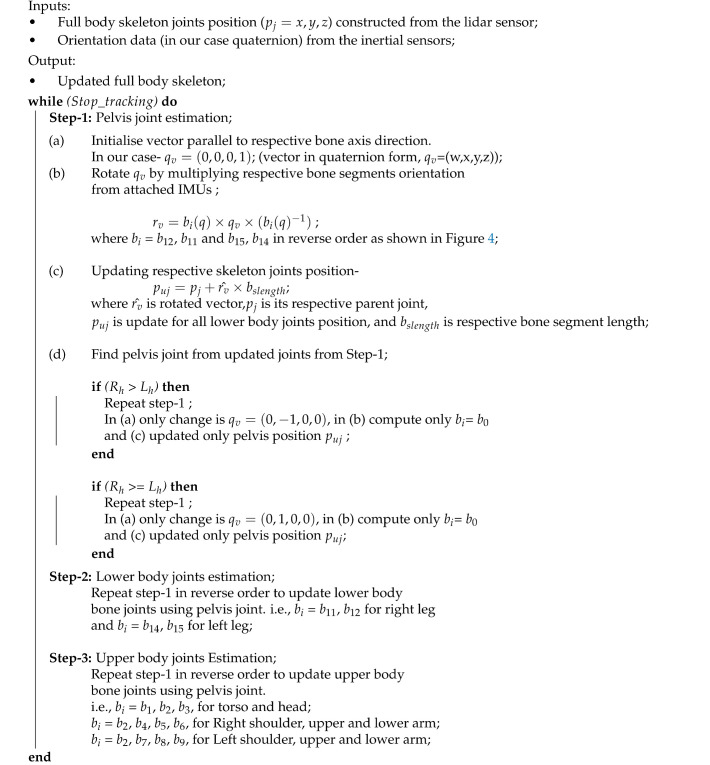


The skeleton base joint (pelvis) position was tracked from the point cloud using the Passthrough filter algorithm [34]. The estimated position from the point cloud was fused with the estimated position of the IMU sensor to correct occlusion and drift over time. The synchronization of sensor data is of prime importance [35]. The data from the IMU sensors are acquired and subjected to calculation at the rate of 60 frames per second, while the data from the lidars are at the rate of 10 frames per second. Therefore, the data are not naturally synchronised. However, since the lidar data are used to constantly compensate the drift in IMU, the compensation is applied by interpolating 6 frames of lidar data against IMU frames. The final estimated position and orientation were then reconstructed on the 3D avatar to visualize real-time tracked human motion.

## 4. Evaluation

This section discusses experimental evaluations to verify the proposed multi-sensor-based human motion tracking system feasibility and robustness. We conducted experimental evaluation as use cases, namely skeleton construction and height accuracy, real-time human tracking and reconstruction. Further, we demonstrate its feasibility and effectiveness by comparing proposed multi-sensor pose tracking with other multiple fusion methods.

### 4.1. Experimental Setup

**Figure 6:**
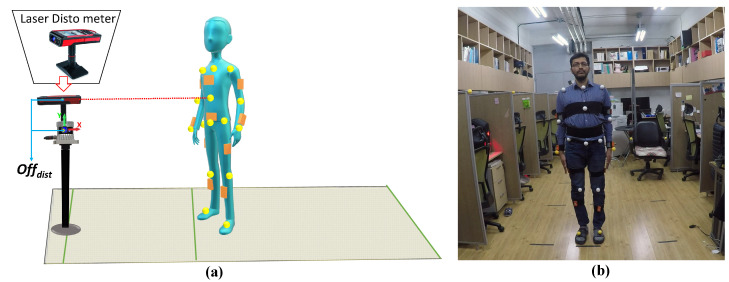
Experimental setup: (**a**) heterogeneous multi-sensor and Leica Disto meter setup for accuracy evaluation, and (**b**) Indoor experimental setup.

Figure 6 shows the experimental setup. We captured user pose ground truth data using a Leica Disto meter [36] from markers placed on bone joints (Figure 6b) simultaneously with data from the proposed heterogeneous multi-sensor tracking system.

We employed Ouster OS-0 lidar and Xsens IMU sensors for the proposed heterogeneous multi-sensor framework. OS-0 offers 32-channel laser scanning with ±90° vertical and 360° horizontal FoV. The sensor scans the environment in 3D at 10 or 20 Hz with multiple horizontal resolution options (512, 1024, and 2048), generating 1,310,720 points per second with range is 55 m. Lidar accuracy ±1.5–5 cm, whereas the Disto meter generates a single-shot 3D position with accuracy ±0.2 cm and range up to 300 m. The Disto meter also provides desktop software [37] to transfer and visualize captured 3D point positions. We considered Disto meter data as ground truth due to its greater accuracy.

Figure 6a shows that the offset distance between Disto meter and lidar position is compensated in *y* and *x* axis directions $off_{dist}$ by 22 and 14 cm, respectively, since generated skeleton bone joint positions were estimated at the human body’s coronal plane, whereas the Disto meter provides 3D positions for markers placed above the human body surface.

Xsens MTW IMU sensors were used to capture bone segment orientations. These are small, lightweight, and wireless inertial sensor 3D motion trackers manufactured using MEMS technology, returning 3D orientation, acceleration, angular velocity, static pressure, and ambient magnetic field intensity at 60 Hz. The orientation from the sensors are directly used for the bone rotation estimation, since the sensors have a built-in filtering method and give a dynamic orientation accuracy of 0.75° RMS (Roll/Pitch) and 1.5° RMS (Yaw). The IMU motes themselves are synchronised by the master controller of the commercially available Xsense sensor suite by 10 μs. The proposed work inherits this feature from the Xsense sensor suite without any modifications. IMU sensors offer orientation in the form of quaternions, Euler angles, and Axis-angles. Only 3D orientation in the form of a quaternion was considered for the proposed work. Real-time full body position and orientation were estimated using the 10 IMU sensors attached to human body segments.

The open-source platform Point Cloud Library [34] in C++ was employed to process and visualize point cloud data from the lidar. We developed a 3D avatar model combining multiple parametric ellipsoids using an open-source visualization toolkit [38] in C++ to visualize tracked human motion on the 3D avatar in real-time. Thus, the proposed heterogeneous multi-sensor system software application was built on an open-source platform and remains open-source.

### 4.2. User Height and Skeleton

To evaluate human pose tracking accuracy, it is important to evaluate the user height and joint position accuracy used to construct the corresponding skeleton structure. Realistic human motion tracking depends strongly on user height bone segment length estimation accuracy. User height was estimated following the proposed method in Section 3.2 at different distances and compared against known user height (ground truth). Table 1 shows user height estimation had mean error ± 1.58 cm, considering inherent lidar sensor error.

**Figure 7:**
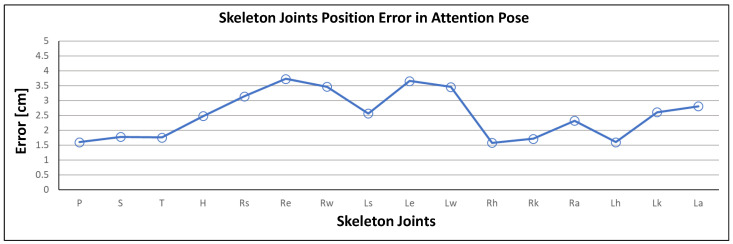
Skeleton reconstruction accuracy from lidar data against ground truth in attention pose.

Skeleton reconstruction accuracy using the estimated height was evaluated for single attention pose joint positions (Figure 6b). Head height was considered as the standard measurement proportion for skeleton joint construction. Figure 7 shows that individual joint position errors compared with ground truth were always <4 cm, with mean error in the attention pose <2.5 cm. Therefore, mean height and skeleton joint position error were insignificant and, therefore, had a minimal effect on human pose tracking.

### 4.3. Pose Tracking and Reconstruction

**Figure 8:**
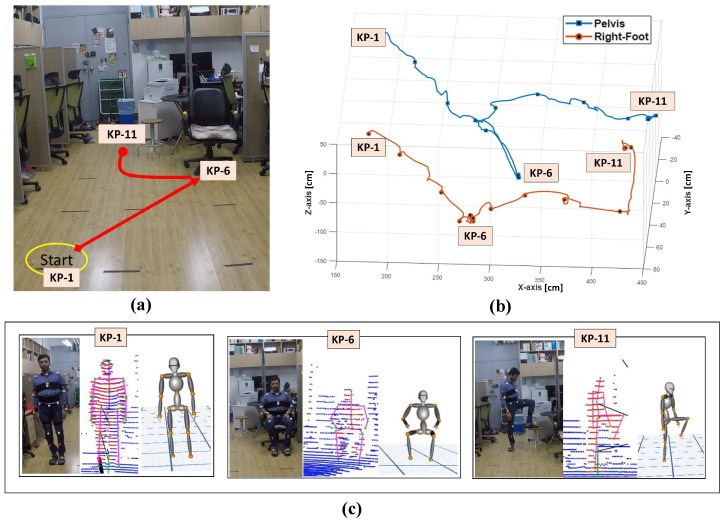
(**a**) Activity scenario for human key pose tracking, (**b**) pelvis and right foot position trajectory tracked using the proposed system, (**c**) real-time user key poses reconstructed on 3D Avatar.

We evaluated the proposed human pose tracking method performance against ground truth data from the Disto meter as discussed in Section 4.1. Figure 8a shows that the user performed an activity requiring significant position movement in the pelvis and right leg for this evaluation: walking from the start position (KP-1), sitting on the chair (KP-6), and then moving to a small table (KP-11) and placing their right foot on the table. We checked the proposed method accuracy against ground truth for two joints: pelvis and right foot position for this scenario.

**Figure 9:**
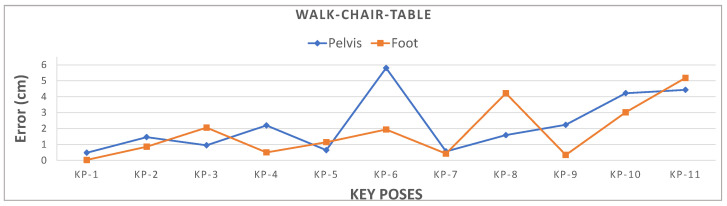
Accuracy for selected pelvis and right foot key poses.

Figure 8b depicts the key poses and continuous frame positions tracked using the proposed method. The position estimation error, i.e., RMSE relative to ground truth, for the pelvis is 2.82 cm and foot is 2.42 cm, which are both well within 3 cm. Figure 9 shows that pelvis Euclidean distance error at KP-6 and right foot position at KP-11 exceed 5 cm due to significant positional movement.

### 4.4. Comparison with Other Methods

**Figure 10:**
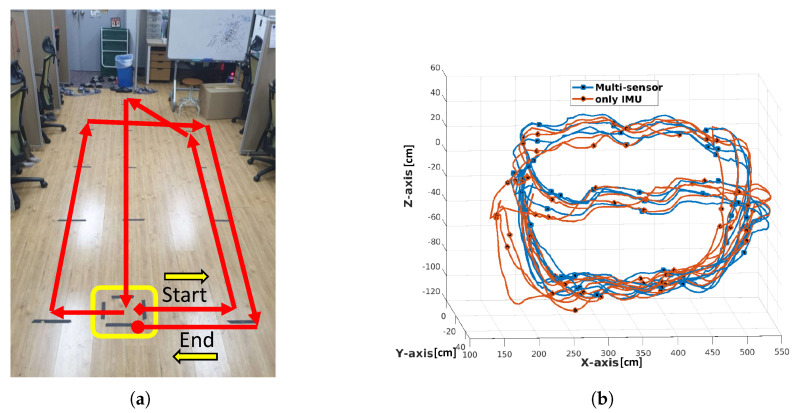
(**a**) Closed loop trajectory path, (**b**) inertial measurement units (IMU) only and multi-sensor system position data trajectory.

This section compares the proposed method with similar work using closed-loop position tracking for drift error analysis. Figure 10a shows the closed-loop path considered to analyze drift error in pelvis joint position. One complete loop from start to end position comprises ∼36 m, and we tracked continuously for four rounds (Figure 10b), i.e., ∼145 m. We recorded the position value after each round, and the ground truth was computed at the start position during initial calibration. Figure 10b shows position drift in IMU tracked data increased in the 3rd and 4th trails.

Table 2 compares Euclidean drift error for IMU-only and proposed multi-sensor tracking against ground truth. IMU-only tracking achieved RMSE drift =32.25 cm, whereas proposed multi-sensor tracking achieved RMSE drift =10.75 cm, which is well within the acceptable range.

Ziegler et al. [2] used a similar lidar and IMU sensor combination for human position tracking in an outdoor environment, and achieved drift error <20 cm for a single 300 m loop. Li et al. [21] used optical and IMU sensors to track human motion in an indoor environment, tracking five trials for IMU-only and fused data. They compared absolute position drift between IMU-only and fused data without ground truth, achieving ∼120 cm in the *x* and *z* axis directions and ∼2 cm in the *y* axis direction. These results are reasonably consistent with the outcomes reported here. Table 3 compares the proposed system setup with those used by Ziegler et al. and Li et al.

**Figure 11:**
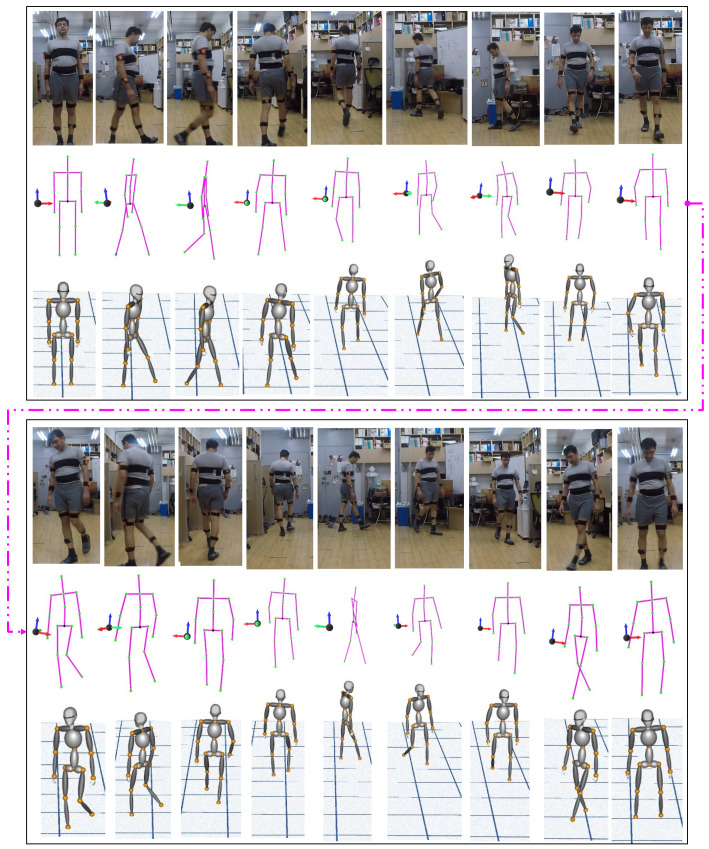
Human pose reconstruction on 3D avatar.

Figure 11 shows the closed-loop walking single trail data (Figure 10) reconstructed in real-time on the virtual 3D avatar, constructed as a combination of multiple parametric ellipsoids using VTK. The reconstruction is realistic and reasonably accurate.

Further, to verify the experimental setup of the proposed system in this paper, we compare it with the other two [2,21] multi-sensor experimental setup. A simple summary of system setup is shown in Table 3.

Finally, to demonstrate the reconstruction accuracy of human motion tracking on a virtual 3D avatar. The results from closed-loop walking single trail (as shown in Figure 10) data are reconstructed in real-time on the virtual 3D avatar. As mentioned earlier, the avatar model built using a combination of multiple parametric ellipsoids using VTK. The sequence of walking steps reconstructed on the 3D avatar demonstrated in Figure 11. The results show that the reconstruction is realistic and reasonably accurate.

## 5. Discussion and Conclusions

The experimental setup for the proposed system was more feasible and flexible concerning sensor locations than previous approaches. Consequently, human pose tracking with heterogeneous multi-sensors was reasonably accurate and within the acceptable range. The proposed multi-sensor system achieved better estimated height and joint position accuracy, and overall better human motion tracking. Preliminary work achieved position tracking accuracy ±3–5 cm using two perpendicular lidars, and 10 IMU sensors; whereas the proposed system achieved accuracy <±3 cm using a single lidar and similar number of IMU sensors. Height and skeleton estimation was minimized and improved using the single lidar sensor due to improved horizontal resolution and vertical FoV.

We used a simple calibration where the user started from an attention position for both lidar and IMU. Skeleton construction enabled automatic derivation for different human height sizes, and vector-based position estimation helped estimate pelvis position using lower body orientation, which would be an effective approach for many applications. Motion reconstruction on the 3D avatar was realistic, due to pelvis position being continuously corrected for occlusion and drift using lidar data. The proposed system could be adopted for real-time pose-tracking applications, such as human–computer interaction, activity recognition, virtual reality, fitness training, healthcare, and rehabilitation. We selected open-source and freely available software and platforms to allow users to use and modify the code.

Future studies will consider ways to improve the current system, including tracking multiple human motions in real-time, estimating independent bone segment movements (e.g., head, shoulder, and both hip bone segments) without sensors attached, by considering orientations from parent bone segment data. More accurate joint position tracking could be achieved by tracking each absolute joint position with the lidar.

## Figures and Tables

**Table 1:** User height estimate accuracy from lidar data at different distances.

Sl. No.	1	2	3	4	5	6	7	8	9	10	11	12
**Distance (m)**	1.25	1.51	1.75	2.04	2.28	2.54	2.90	3.20	3.82	4.50	5.35	5.92
**Height (cm)**	171	170	169	173	174	175	166	168	171	167	173	168
**Mean error (cm)**	1.58											

**Table 2:** Drift error for IMU-only and proposed multi-sensor system against ground truth position.

Trails	IMU Only (cm)	Multi-Sensor (cm)
1	6.4339	9.1725
2	13.9382	12.5533
3	43.0608	11.6600
4	45.4884	9.1796

**Table 3:** Comparison of human motion tracking system setup.

	Li et al. [21]	Zielger et al. [2]	Our Proposed System
**Position tracking** **sensor**	HTC VIVE 2 Base station 6 Trackers	Mobile robot equipped with SICK LMS laser ranger	Ouster OS0 Lidar sensor
**Inertial sensor**	Perception Neuron 17 IMUs	Xsens MVN 17 IMUs	Xsens Awinda 10 IMUs
**Experiment setup**	Indoor Environment	Outdoor Environment	Indoor Environment
**3D model**	Self developed Skeleton model	Xsens provided Skeleton model	Self developed Skeleton and Avatar model
**Open-source** **platform**	No	No	Yes
**Drift accuracy**	∼120 cm in x and z ∼2 cm in y	<20 cm	<11 cm

## Data Availability

Data is contained within the article.

## Abbreviations

The following abbreviations are used in this manuscript:

lidar	Light Detection and Ranging
IMU	Inertial Measurement Unit
FoV	Field of View
2D	Two-dimensional
3D	Three-dimensional
VTK	Visualization Tool Kit
RMSE	Root Mean Square Error
KP	Key Pose
